# Is IMRT Superior or Inferior to 3DCRT in Radiotherapy for NSCLC? A Meta-Analysis

**DOI:** 10.1371/journal.pone.0151988

**Published:** 2016-04-21

**Authors:** Xingsheng Hu, Wenwu He, Shimin Wen, Xuqin Feng, Xi Fu, Yusong Liu, Ke Pu

**Affiliations:** 1 Department of Oncology, Nanchong Central Hospital (the Second Affiliated Hospital of North Sichuan Medical College), Nanchong, Sichuan Province, China; 2 Department of Cardiothoracic surgery, Nanchong Central Hospital, Nanchong, Sichuan Province, China; 3 Department of Clinical Laboratory, The People's Hospital of Shifang, Shifang, Sichuan Province, China; 4 DaZhou college of Chinese medicine, DaZhou, Sichuan Province, China; Univesity of Texas Southwestern Medical Center at Dallas, UNITED STATES

## Abstract

**Introduction:**

There are no adequate data to determine whether intensity-modulated radiotherapy (IMRT) is superior to three-dimensional conformal radiotherapy (3DCRT) in the treatment of non-small cell lung cancer (NSCLC). This meta-analysis was conducted to compare the clinical outcomes of IMRT and 3DCRT in the treatment of NSCLC.

**Methods:**

No exclusions were made based on types of study design. We performed a literature search in PubMed, EMBASE and the Cochrane library databases from their inceptions to April 30, 2015. The overall survival (OS) and relative risk (RR) of radiation pneumonitis and radiation oesophagitis were evaluated. Two authors independently assessed the methodological quality and extracted data. Publication bias was evaluated by funnel plot using Egger’s test results.

**Results:**

From the literature search, 10 retrospective studies were collected, and of those, 5 (12,896 patients) were selected for OS analysis, 4 (981 patients) were selected for radiation pneumonitis analysis, and 4 (1339 patients) were selected for radiation oesophagitis analysis. Cox multivariate proportional hazards models revealed that 3DCRT and IMRT had similar OS (HR = 0.96, P = 0.477) but that IMRT reduced the incidence of grade 2 radiation pneumonitis (RR = 0.74, P = 0.009) and increased the incidence of grade 3 radiation oesophagitis (RR = 2.47, P = 0.000).

**Conclusions:**

OS of IMRT for NSCLC is not inferior to that of 3DCRT, but IMRT significantly reduces the risk of radiation pneumonitis and increases the risk of radiation oesophagitis compared to 3DCRT.

## Introduction

Radiotherapy is one of the most effective treatments for cancer. Among cancer treatments, surgery accounts for 22%, radiotherapy for 18%, and chemotherapy for 5%. Radiotherapy remains an important component of passive therapy. Lung cancer is the leading cancer in both incidence and mortality and accounts for 25% of all cancer deaths, and the incidence of lung cancer continues to rise. At least 50% of patients with locally advanced non-small cell lung cancer (NSCLC) receive radiotherapy [1].

Several important advanced techniques in radiotherapy treatment have emerged in the past few decades that are changing the picture of cancer treatment. These advanced techniques include three-dimensional conformal radiotherapy (3DCRT), intensity-modulated radiotherapy (IMRT), image-guided radiotherapy (IGRT), stereotactic body irradiation, tomotherapy, and proton and particle beam therapy [2]. The main goals of these radiotherapy techniques are (1) to increase the conformal degree of the target area and increase the radiation dose, and (2) to decrease toxicity to normal tissue to improve locoregional control (LRC) and overall survival (OS). 3DCRT was approved in the 1990s to reduce toxicity to normal tissue and increase LRC and OS compared to 2DCRT. IMRT is more effective than 3CRT in enabling conformal radiation and increased dose [3] and reducing toxicity to normal tissue [4–5]. This advantage of IMRT is important, particularly for tumours located close to regions such as the spinal cord, brain stem, and eyes. Recent accumulating evidence supports the use of IMRT in prostate [6] and head and neck cancers [7] based on the reduction of treatment toxicity. However, the use of IMRT in locally advanced lung cancers remains controversial. The main concerns stem from two potential areas: (1) IMRT increases the amount of normal lung tissue exposed to low doses of radiation and could potentially increase the risk of radiation pneumonitis and (2) lung tumours move due to the interplay of breathing, the motion of the tumour, and the motion of multileaf collimator(MLC)-shaped segments, potentially inducing unanticipated variations of the dose delivered to the target [8–9]. Data or randomized trials to provide clear guidance for clinical practice are lacking. Thus, we performed this meta-analysis to evaluate the outcomes of IMRT compared to 3DCRT in terms of survival and toxicity.

## Materials and Methods

### Study design

This meta-analysis followed PRISMA statement guidelines [10].

### Eligibility criteria

The following study selection criteria were applied. (1) No exclusions were made based on the type of study design. (2) The diagnosis of NSCLC was confirmed by pathological study. (3) An association between IMRT and 3DCRT and outcomes in the treatment of NSCLC was demonstrated. (4) The study included at least 20 cases. (5) The hazard ratios (HR) and the corresponding 95% confidence intervals (CIs) of OS, the incidence of radiation pneumonitis or oesophagitis, or the number of cases of radiation pneumonitis or oesophagitis could be obtained.

### Literature search

We conducted a comprehensive literature review in PubMed, Embase and Cochrane Library from inception to April 30, 2015 and identified articles that evaluated the associations between IMRT and 3DCRT in the treatment of NSCLC. The key words were intensity-modulated radiation therapy (IMRT) and conformal radiation therapy (3DCRT) and non-small cell lung cancer (NSCLC). No language restrictions were imposed. In addition, we screened references from retrieved original articles to identify other potentially eligible studies.

### Study selection and quality assessment

Two reviewers independently assessed the methodological quality of the included studies using the Newcastle–Ottawa Scale (NOS) [11]. The NOS contains three parameters of quality: selection, comparability, and exposure assessment. The NOS assigns maximum scores of 4 for selection, 2 for comparability, and 3 for exposure. Hence, a score of 9 indicates the highest quality. Discrepancies were addressed in consultation with a third reviewer. We considered studies with scores of 6 or higher as high-quality studies.

### Data extraction

Two reviewers independently extracted the following data from each eligible study: first author’s last name, year of publication, number of patients, ages, region, stage, radiotherapy (RT) dose, use of CCRT, HR of OS, and incidence and number of cases of radiation pneumonitis or oesophagitis. Any disagreements were resolved by consensus or in consultation with a third reviewer.

### Statistical analysis

We estimated the HR and 95% confidence interval (CI) for OS and the relative risk (RR) of the incidence of radiation pneumonitis and oesophagitis. Heterogeneity was determined using the chi-square based Cochran’s Q statistic and the I^2^ statistic. The I^2^ yield results ranged from 0 to 100% (I^2^ = 0–40%, low heterogeneity; I^2^ = 40–60%, moderate heterogeneity; I^2^ = 50–90%, large heterogeneity; and I^2^ = 75–100%, extreme heterogeneity) [12]. If large heterogeneity existed, the random-effects model was used; otherwise, the fixed-effects model was used. If significant heterogeneity was identified, subgroup analysis was conducted. Sensitivity analyses were conducted to test the robustness of the main results by removing each study in turn. Potential publication bias was evaluated by funnel plots, and Egger’s weighted linear regression test was used to examine the asymmetry of the funnel plots [13]. All statistical analyses were performed using Stata 12.0. All P values were two-sided and were considered significant at the .05 level.

## Results

### Study selection and characteristics

**Fig 1:**
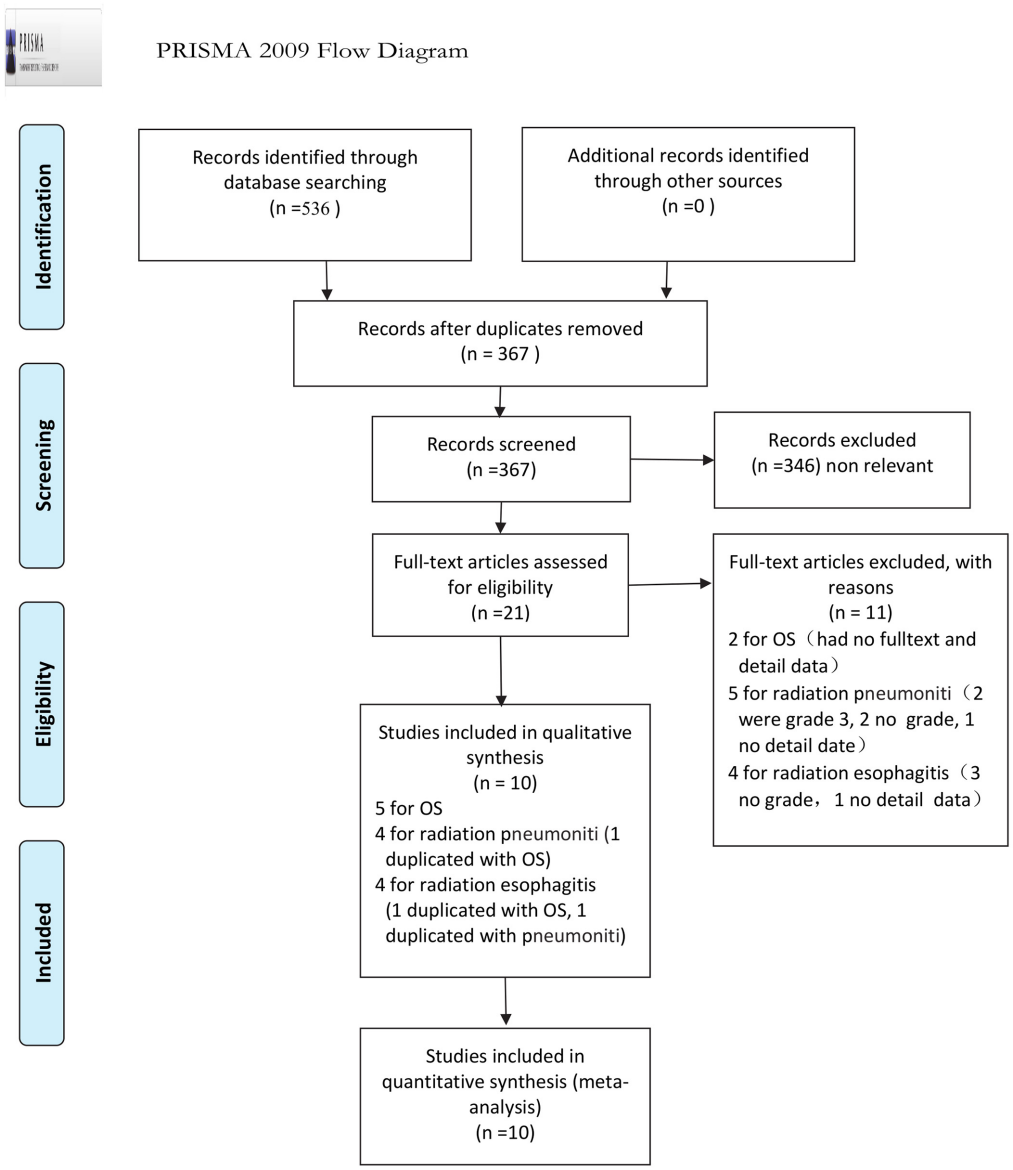
PRISMA Flow chart of the search result of the meta-analysis. *From*: Moher D, Liberati A, Tetzlaff J, Altman DG, The PRISMA Group (2009). *P*referred *Reporting I*tems for *S*ystematic Reviews and *M*eta-*A*nalyses: The PRISMA Statement. PLoS Med 6(6): e1000097. doi: 10.1371/journal.pmed1000097. For more information, visit www.prisma-statement.org

A total of 536 relevant studies were identified during the initial search. Ultimately, 5 studies (12,896 patients) were included in the OS meta-analysis, 4 studies (two abstracts, 981 patients) were included in the radiation pneumonitis meta-analysis, and 4 studies (one abstract, 1339 patients) were included in the radiation oesophagitis meta-analysis. All studies were retrospective research. The flow diagram of the literature retrieval and selection is presented in Fig 1.

The main characteristics of all eligible studies are presented in Tables 1, 2, 3, 4, 5 and 6 present the incidence of radiation pneumonitis and radiation oesophagitis, respectively. The studies were published between 2010 and 2015. With the exception of three abstracts, the studies provided comprehensive clinical information. The mean age of the subjects in the studies was approximately 65 years old. Geographically, 7 studies were conducted in the USA, 1 in Taiwan, and 1 in Korea; 2 abstracts did not provide regional information. Stage III cancer was the most common stage in the studies, and the mean dose was approximately 60 Gy, including both concurrent chemoradiation (CCRT) and sequential chemoradiotherapy (SCRT).

**Table 1:** Main characteristics of the studies for OS.

Study	Pts	Age (years)	region	Stage	RT dose	CCRT or SCRT
Liao ZX2010[14]	409	unlimited	USA	Unresectable pts	mean 63Gy (50–73)	CCRT
Harris JP 2014[15]	6894	≥65	USA	III	RT length (3-9weeks)	CCRT, SCRT
Hsia TC 2014[16]	99	unlimited	Taiwan	III	≥50Gy	CCRT, SCRT
Chen AB 2014[17]	5417	≥65	USA	III	>25fractions (>45–50 Gy)	NG
Noh JM 2015[18]	77	median60 (40–80)	Korea	III B	mean 66Gy	CCRT

Pts: patients, RT: radiotherapy, CCRT: concurrent chemoradiation, SCRT: sequential chemoradiotherapy, NG: not give.

**Table 2:** Main characteristics of the studies for OS.

Study	HR (95%C I) of OS in univariate	HR (95%C I) of OS in Cox multivariate	HR (95%C I) of OS not limited to curative RT dose
Liao ZX 2010	NG	0.64 (0.41–0.98) P = 0.039	NG
Harris JP 2014	0.90 (0.82–0.98) P = 0.02	0.94 (0.85–1.04) P = 0.23	0.94 (0.85–1.04) P = 0.23
Hsia TC 2014	NG	1.54 (0.82–2.91) P = 0.18	NG
Chen AB 2014	0.95 (0.87–1.0) P = 0.24	0.99 (0.9–1.09) P = 0.82	0.89 (0.84–0.99) P = 0.03
Noh JM 2015	NG	2.16 (0.67–6.96) P = 0.197	NG

**Table 3:** Main characteristics of the studies for radiation pneumonitis.

Study	Pts	Age (years)	region	Stage	RT dose (Gy)	CCRT or not
Sejpa S 2011[19]	140	median 61 (38–82)	USA	III	mean 63	CCRT
McCloskey	424	NA	Canada	III	mean 61 (3DCRT)	74% (3CDRT) and 78% (IMRT)
2012(abstract) [20]					mean 66 (IMRT)	were CCRT
Rehman S	340	median 65	NA	NA	NA	NA
2014(abstract) [21]						
Noh JM 2015[18]	77	median 60 (40–80)	Korea	IIIB	mean 66Gy	CCRT

Pts: patients, RT: radiotherapy, CCRT: concurrent chemoradiation, SCRT: Sequential chemoradiotherapy, NA: not obtained.

**Table 4:** The incidence of radiation pneumonitis.

study	radiation methods	positive (n)	negative (n)	rate
Sejpa S 2011	IMRT	23	43	9%
	3D-CRT	44	30	30%
McCloskey 2012	IMRT	19	88	18%
	3D-CRT	51	166	24%
Rehman S 2014	IMRT	20	43	32%
	3D-CRT	97	180	35%
Noh 2015	IMRT	7	22	24%
	3D-CRT	16	32	33%

**Table 5:** Main characteristics of the studies for radiation esophagitis.

Study	Pts	Age (years)	region	Stage	RT dose (Gy)	CCRT or not
Gomez D 2011 [22]	678	NA	NA	I-IV	≥60	72% were CCRT
Sejpa 2011[19]	140	median 61 (38–82)	USA	III almost	mean 63	CCRT
Gomez DR 2012[23]	444	median 66 (33–92)	USA	I-IV (III almost)	mean 63 (50–87.5)	CCRT or not
Noh JM 2015[18]	77	median 60 (40–80)	Korea	III B	mean 66	CCRT

Pts: patients, RT: radiotherapy, CCRT: concurrent chemoradiation,SCRT: Sequential chemoradiotherapy, NA: not obtained.

**Table 6:** The incidence of radiation esophagitis.

study	radiation methods	positive (n)	negative (n)	rate
Gomez D 2011	IMRT	33	89	27%
	3D-CRT	65	397	14%
Sejpa S 2011	IMRT	29	37	44%
	3D-CRT	13	61	18%
Gomez DR 2012	IMRT	39	100	28%
	3D-CRT	32	373	8%
Noh 2015	IMRT	8	21	27%
	3D-CRT	7	41	15%

### Quality of the included studies

The quality assessments of the evaluated studies are presented in Table 7 (OS), Table 8 (radiation pneumonitis) and Table 9 (radiation oesophagitis). All studies were determined to be of high quality (NOS score greater than 6). The most common selection biases were the selection of controls from hospital controls and the definition of controls. In terms of comparability bias, the most common factors were tumour volume, comorbidity, nodal stage, RT dose, CCRT/SCRT, PET scans, and brain imaging. All studies had good ascertainment of exposure and did not indicate a non-response rate.

**Table 7:** The NOS score of studies for OS.

study	section				Comparability	Exposure			total
	Is the case definition adequate?	Representativeness of the Cases	Selection of Controls	Definition of Controls		Ascertainment of Exposure	Is same method for case and control?	Nonresponse rate	
Liao ZX 2010	1	1	0	0	1	1	1	1	6
Harris JP 2014	1	1	1	1	1	1	1	1	8
Hsia TC 2014	1	1	1	1	2	1	1	1	9
Chen AB 2014	1	1	1	1	2	1	1	1	9
Noh JM 2015	1	1	0	0	1	1	1	1	6

**Table 8:** The NOS score of studies for radiation pneumonitis.

study	section				Comparability	exposure			total
	Is the case definition adequate?	Representativeness of the Cases	Selection of Controls	Definition of Controls		Ascertainment of Exposure	Is same method for case and control?	Nonresponse rate	
Sejpal S 2011	1	1	0	0	1	1	1	1	6
Noh JM 2015	1	1	0	0	1	1	1	1	6

**Table 9:** The NOS score of studies for radiation esophagitis.

study	section				Comparability	exposure			total
	Is the case definition adequate?	Representativeness of the Cases	Selection of Controls	Definition of Controls		Ascertainment of Exposure	Is same method for case and control?	Nonresponse rate	
Sejpal S 2011	1	1	0	0	1	1	1	1	6
Gomez DR 2012	1	1	0	0	1	1	1	1	6
Noh JM 2015	1	1	0	0	1	1	1	1	6

### OS

#### Univariate

**Fig 2:**
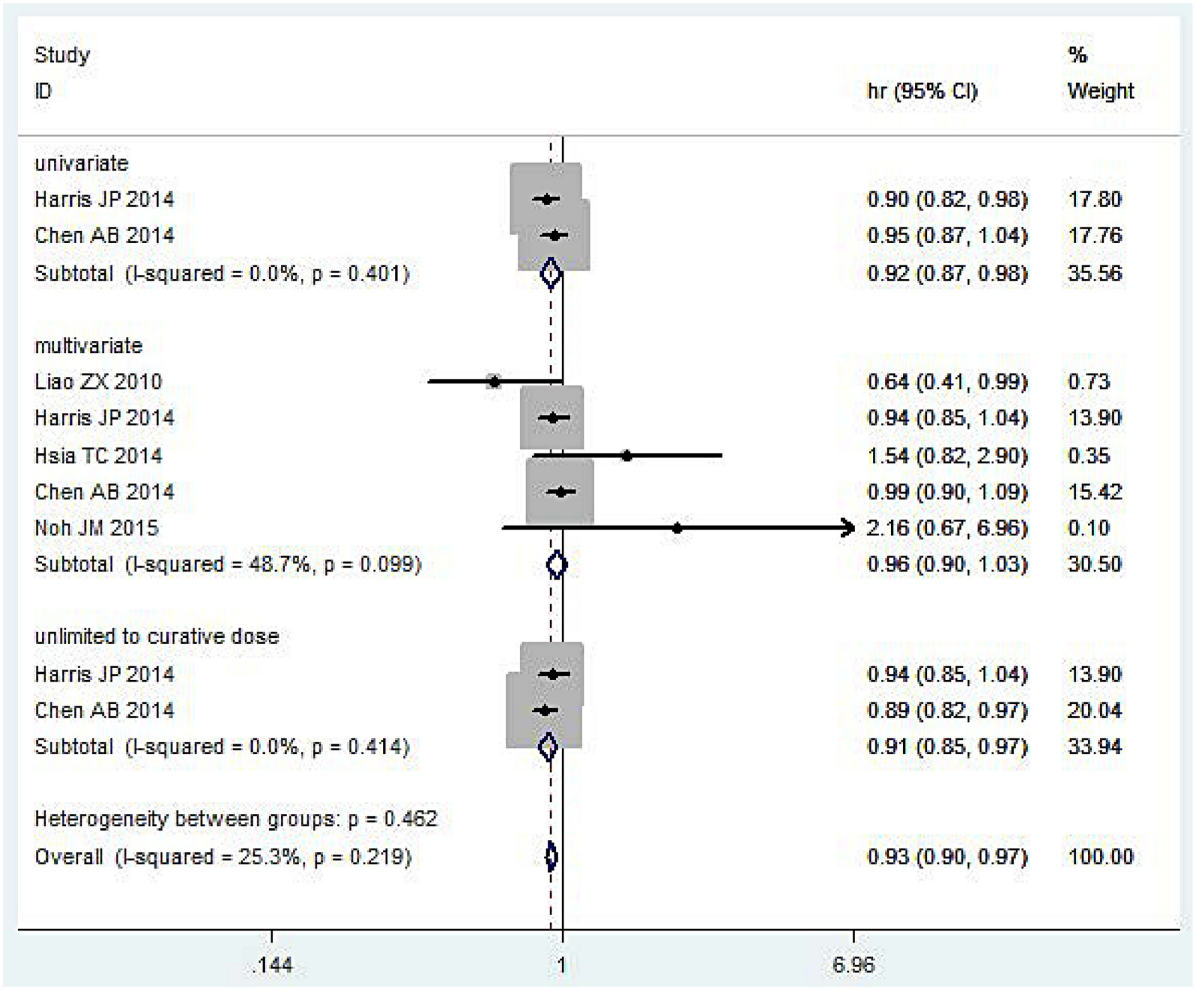
Meta-analysis result of OS.

Two studies [15, 17**]** (12,311 patients) of the HR of OS determined by univariate analysis were incorporated into this meta-analysis (Fig 2). The pooled results indicated that IMRT reduced the mortality risk of OS (HR = 0.92, 95%CI: 0.87–0.98, P = 0.015). No significant heterogeneity was detected (I^2^ = 0.00%, P = 0.401).

#### Multivariate

A total of 5 studies [14–18] (12,896 patients) of the HR of OS determined by Cox multivariate analysis were incorporated into this meta-analysis (Fig 2). The pooled results indicated that there was no significant difference between IMRT and 3DCRT (HR = 0.96, 95%CI: 0.90–1.03, P = 0.477). No significant heterogeneity was detected (I^2^ = 49.1%, P = 0.097).

#### Not limited to curative RT dose

Two studies [15, 17] (12,311 patients) of patients not limited to curative RT doses for OS were incorporated into this meta-analysis (Fig 2). The pooled results indicated that IMRT improved OS (HR = 0.91, 95%CI: 0.85–0.97, P = 0.004). No significant heterogeneity was detected (I^2^ = 0.00%, P = 0.410).

### Toxicity

#### Radiation pneumonitis

**Fig 3:**
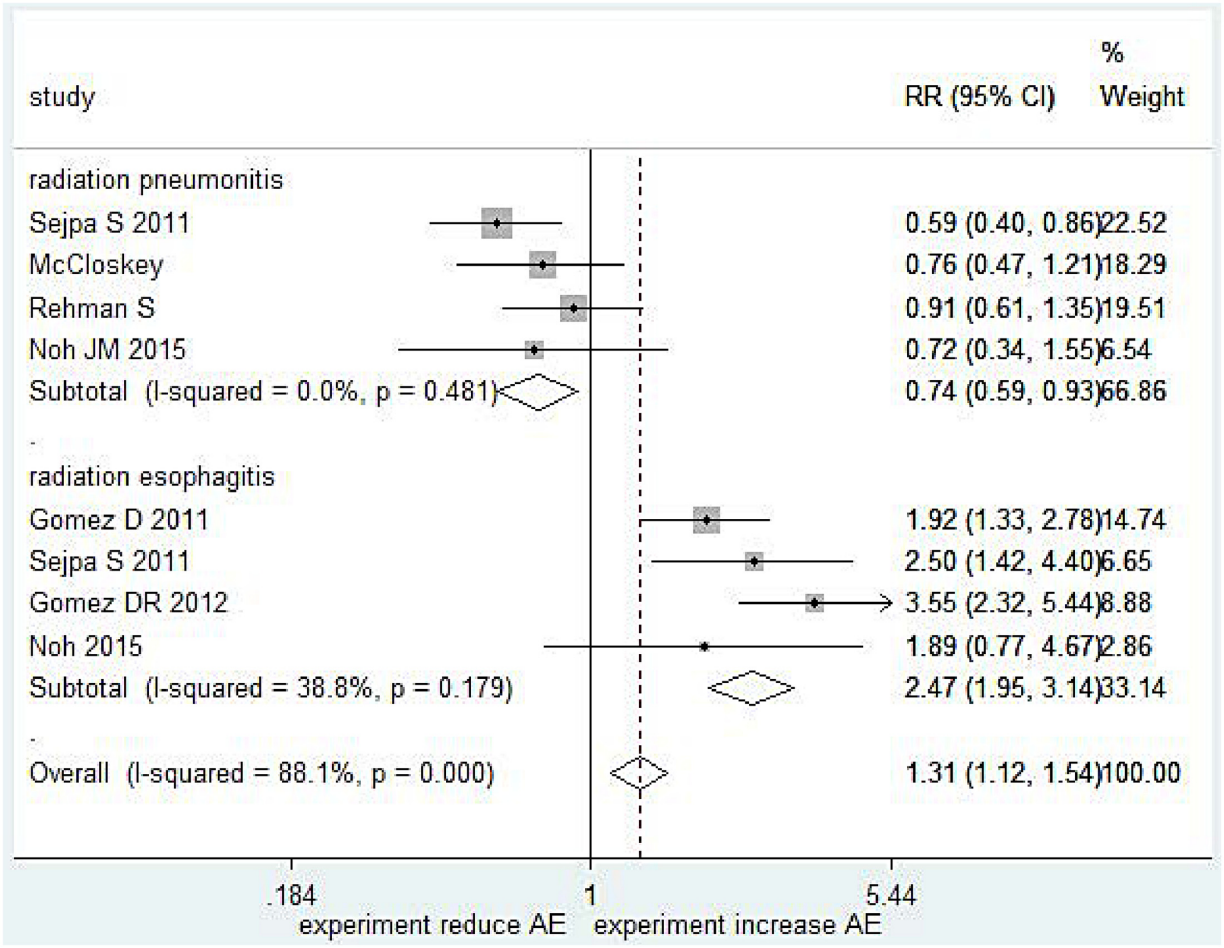
Meta-analysis result of radiation pneumonitis and radiation esophagitis.

Four studies [18–21] (557 patients, including 2 abstracts) of the incidence of grade 2 radiation pneumonitis were incorporated into this meta-analysis (Fig 3). The pooled results indicated that IMRT reduced the incidence of grade 2 pneumonitis (RR = 0.74, 95%CI: 0.59–0.93, P = 0.009). No significant heterogeneity was detected (I^2^ = 0.0%, P = 0.481).

#### Radiation oesophagitis

Four studies [18–19, 22–23] (1337 patients, include 1 abstract) regarding the incidence of grade 3 radiation oesophagitis were incorporated into this meta-analysis (Fig 3). The pooled results indicated that IMRT increased the incidence of grade 3 oesophagitis (RR = 2.47, 95%CI: 1.95–3.14, P = 0.000). No significant heterogeneity was detected (I^2^ = 38.89%, P = 0.129).

#### Publication bias

**Fig 4:**
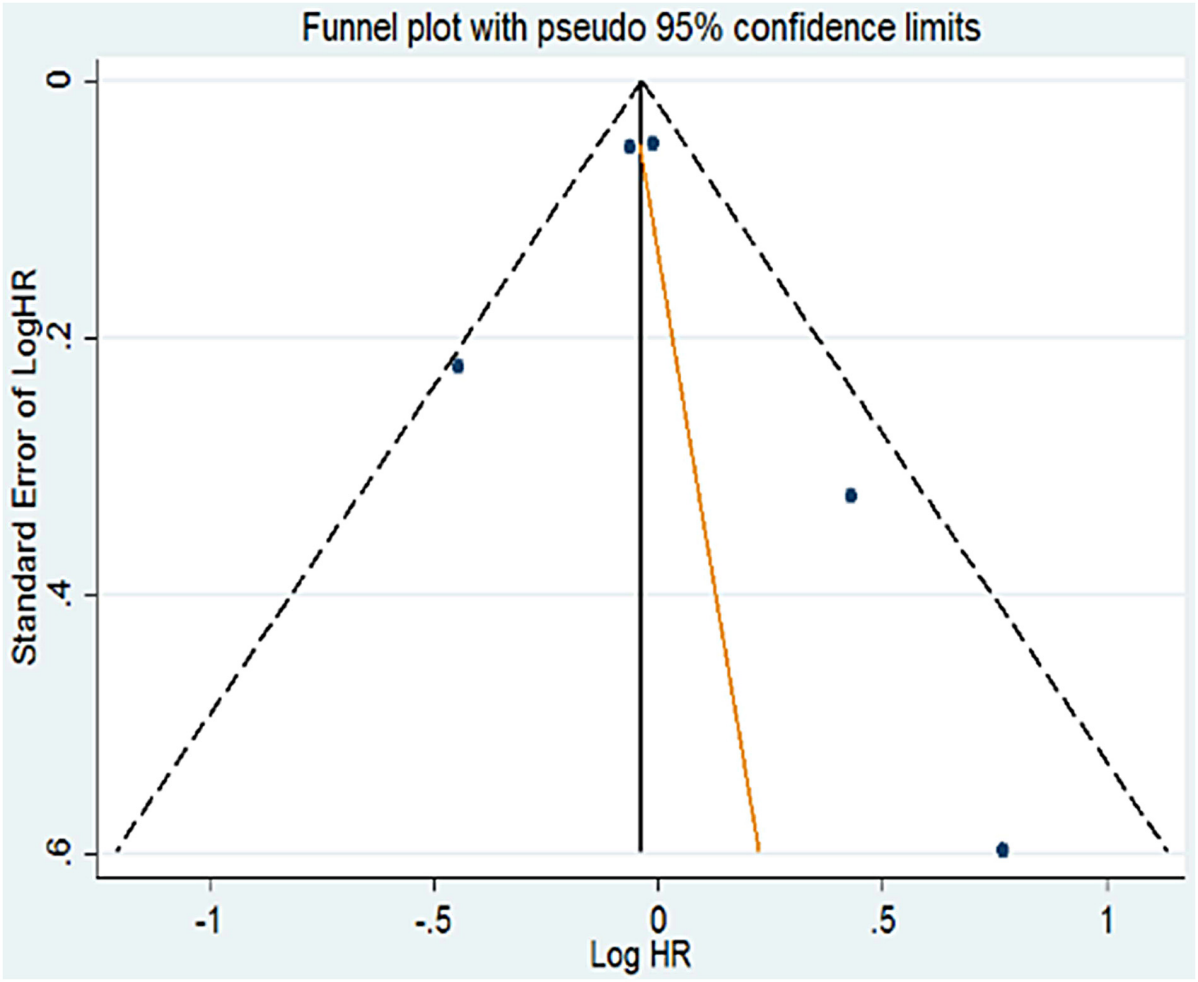
Funnel plot based on odds ratio for OS in multivariate analysis.

Funnel plots and Egger's test were used to explore publication bias. Fig 4 show the Funnel plots of OS. No significant publication bias was identified (Egger’s test: P = 0.686 for multivariate analysis of OS; P = 0.915 for radiation pneumonitis; P = 0.940 for radiation oesophagitis).

#### Sensitivity analysis

Sensitivity analysis was conducted to explore the impact of one study on the pooled results in OS, radiation pneumonitis and radiation oesophagitis. These results indicate that the main results were robust to the removal of one study in turn.

## Discussion

### Key findings and clinical associations

#### OS

Based on original study data, we categorized the HR of the OS analyses into univariate, multivariate, and not limited to curative radiotherapy dose analyses and focused on the multivariate analysis. According to the univariate analysis model, OS was significantly improved in the IMRT group, with a reduction of the risk of death of 7.5% (HR = 0.92, P = 0.015). In the multivariate analysis model, there was no significant difference between the IMRT and 3DCRT groups (HR = 0.96, P = 0.477). The study by Noh [18] included more significant adverse prognostic factors (supraclavicular lymph node metastasis and larger tumour volume), and thus we omitted this study. Thus, the pooled HR was 0.961 (95%CI: 0.898–1.029, P = 0.259) but the main result did not change. Because Noh [18] included patients not limited to curative radiotherapy, the OS was significantly improved in the IMRT group. In Harris’s study [15], the length of radiotherapy treatment ranged from 3 to 9 weeks, and thus this study was not limited to curative radiotherapy. We combined these two studies, and the results indicated that the OS was significantly improved in the IMRT group, with a decrease in the risk of death of 9% (HR = 0.91, P = 0.004).

Our survival meta-analysis included 12,896 subjects. We assessed the methodological quality of the included studies using the NOS, which showed that the quality of the available studies was generally good. The heterogeneity test indicated that there was no significant heterogeneity, the funnel plot was approximately symmetrical, and Egger’s test detected no significant publication bias. Sensitivity analysis confirmed the stability of the main results. Our main goal was to compare the HRs using the Cox multivariate model to decrease the influence of confounding factors and different follow-up times in the different studies and consequently increase the robustness of the main results. This is the first meta-analysis to compare IMRT and 3DCRT in the treatment of NSCLC. There have also been no prospectively randomized trials comparing the clinical outcomes of IMRT and 3DCRT in the treatment of NSCLC. Hence, no rigorous evidence exists to help determine whether IMRT is superior to 3DCRT in thoracic radiotherapy. However, the prevalence of IMRT is increasing worldwide. The main concerns about its use are whether IMRT increases the rate of radiation pneumonitis because larger volumes of normal tissue are exposed to low-dose radiation and whether potentially inferior cancer outcomes related to the interplay between MLC and tumour motion exist. Our findings indicate that IMRT is no less effective than 3DCRT in the treatment of NSCLC, and we obtained a similar HR for OS using the Cox multivariate model. In the univariate analysis, IMRT reduced the risk of death in patients with NSCLC, indicating that IMRT has a tendency to influence and improve the outcomes of NSCLC. Thus, IMRT may be a confounding factor but not an independent prognosis factor. In the analysis of patients not limited to curative radiotherapy, IMRT also improved the outcomes of NSCLC, which indicates that depending on the treatment goals, survival time may be marginally longer in IMRT patients than those treated with 3DCRT. IMRT has generally been used for head and neck cancer, and three randomized trials have been conducted. Gustavo [7] conducted a meta-analysis that included 5 prospective phase III randomized trials to compare IMRT and 3DCRT in the treatment of head and neck cancers. The pooled results demonstrated that IMRT was associated with similar locoregional control and OS but had a significant overall benefit in reducing the incidence of grade 2–4 xerostomia (HR = 0.76; P<0.0001). The results of our OS analysis were similar to those of Gustavo. Our findings also indicated that the interplay between MLC and tumour motion did not influence the survival outcomes of NSCLC patients. Prior studies have demonstrated that the effects of interplay between MLC and tumour motion are likely ‘‘washed out” with multiple fields over the course of radiotherapy [24–26]. A phantom study examining the difference in dose between static points and moving target points during step-and-shoot IMRT delivery determined that the intrafractional variation in target coverage was <5% and the maximum mean or median variation was 0.6% [27–28].

### Toxicity

#### Radiation pneumonitis

The incidence of grade 2 radiation pneumonitis was significantly lower in the IMRT group than the 3DCRT group, and IMRT reduced the risk of radiation pneumonitis by 27% (RR = 0.74, P = 0.009). The heterogeneity test, sensitivity analysis, funnel plot and Egger’s test all indicated that the main results were robust and that no significant publication bias existed (P<0.05). Another study [29] did not provide details on the number of cases of radiation pneumonitis, and thus it was not included in our meta-analysis. That study observed a significantly lower incidence of grade 2 pneumonitis in the IMRT group compared to the 3DCRT group (2.5% versus 4.5%, P < .0001). Even after dose escalation, the incidence of grade 2 pneumonitis was significantly lower in the IMRT group than in the 3DCRT group (5.5% versus 6.8%, P < .0001).

Two other studies provided information on grade 3 radiation pneumonitis. Yom [30] observed that the estimated rate of grade 3 radiation pneumonitis was 8% for IMRT patients, compared with 32% for 3DCRT patients (P *=* 0.002). Liao [14] reported that IMRT also statistically significantly decreased the incidence of grade 3 radiation pneumonitis (HR = 0.33, P = 0.017), but that study did not provide details on the number of cases of radiation pneumonitis. Two other studies reported the overall incidence of acute lung toxicity but did not provide the rate of grade 2 radiation pneumonitis. Shervin [31] observed that the overall incidence of acute lung toxicity within 8 months of treatment was 10.5% in the IMRT group and 7.8% in the 3DCRT group (P = 0.12) using the narrow definition and 38.9% (IMRT) and 37.5% (3DCRT) (P = 0.80) using the broad definition. Harris [15] performed multivariate analysis and demonstrated that IMRT was not associated with a significantly different risk of early pulmonary toxicity (HR = 1.14, P = 0.23) or late pulmonary toxicity (HR = 1.22, P = 0.33). IMRT reduces the incidence of radiation pneumonitis, primarily due to its dosimetric advantages in radiotherapy planning [32], which expose larger volumes of normal tissue to low-dose radiation. In most of the studies [14,29,30], the V20 and the mean doses to normal lung tissue were reduced or were at least not higher than in the 3DCRT group, despite the large tumour volumes. V5 increased, indicating that larger volumes of normal tissue were exposed to low-dose radiation; however, V5 did not correlate with a higher incidence of radiation pneumonitis, which indicated that V5 is not a decisional prognosis factor for radiation pneumonitis.

#### Radiation oesophagitis

In contrast to our expectations, we observed that the incidence of acute oesophagus toxicity was significantly higher in the IMRT group than in the 3DCRT group. IMRT generally reduces the incidence of radiation oesophagitis in addition to radiation pneumonitis. However, we observed a higher incidence of radiation oesophagitis in the IMRT group. Four studies with good concordance indicated a higher incidence of acute oesophagus toxicity in the IMRT group than in the 3DCRT group. The pooled results were also significant.

The number of studies indicating this phenomenon are limited, but several potential underlying factors can be proposed. (1) The spatial distribution of the oesophageal dose may have been different for the IMRT group, specifically the cross-sectional anterior-posterior area and/or superior-inferior location of the irradiated region. Thus, the oesophageal area may have received more low-dose radiation [23]. (2) To ensure that the lung and spinal doses are lower than the defined constraints, the oesophageal area may have received more radiation. Lievens [29] observed that when the lung and spinal cord doses were lower than the defined constraints, the oesophageal dose increased significantly in parallel with the increase in the prescribed dose. After dose escalation, acute or late oesophageal toxicity increased consistent with the greater prescription dose. However, when the prescription dose was deescalated to conform to the predefined oesophageal dose limits, the advantage of IMRT was lost. (3) The IMRT group in general may have had large tumour volumes or more lymph node involvement, even in some limited regions (such as the mediastina), and thus the oesophageal area may have received a higher dose. Schwarz [33] found the oesophagus to be the dose-limiting structure in IMRT planning.

However, we remain cautious because only 4 studies were included in this meta-analysis, and we only estimated grade 3 oesophagitis. Three studies did not estimate grade 3 oesophagitis and were not included in our meta-analysis. Shervin [31] calculated the overall incidence of acute oesophagitis and HR. The overall incidence of acute oesophagitis was 44% in the IMRT group and 42% in the 3DCRT group (P = 0.58), HR = 1.06 (P = 0.56). Harris [15] included radiation oesophagitis in a study of upper gastrointestinal toxicity and observed no difference in upper gastrointestinal toxicity between the IMRT and 3DCRT groups. Lievens [29] observed that IMRT significantly decreased acute oesophagitis at low dose levels (p < .0001), but after maximal dose escalation, late oesophageal tolerance increased significantly in parallel with the prescribed dose (34.7% versus 17.3%, p < .0001) compared to 3DCRT.

### Limitations

1. There were no randomized trials or cohort studies that compared IMRT and 3DCRT in the treatment of NSCLC, and thus all studies were retrospective research. Although the authors of the included studies controlled for demographic and clinical characteristics, the potential for confounding by unmeasured attributes may also exist. Few original studies were included in this meta-analysis model: 5 in the OS analysis, 4 in the radiation pneumonitis analysis (including 2 abstracts) and 4 in the radiation oesophagitis analysis (1 was an abstract). Despite several attempts, we were unable to obtain the full text of 3 abstracts because they were published in supplements. In the five studies used for OS analysis, there were no sufficient and accordant data to evaluate toxicity, and thus we searched additional studies for toxicity analyses. In addition, radiation pneumonitis and oesophagitis were categorised in different grades in different original studies, complicating meta-analysis.
2. Except for OS, most of the studies did not analyse PFS or LRC; only one study [18] provided information on PFS. PFS is an important factor in tumour survival analysis; many clinical trials have observed significant differences in PFS but not OS.
3. Most of the included studies did not perform sufficient subgroup analysis, particularly for several important subgroups such as CCRT/SCRT, targeting therapy, and tumour size. Only 2 studies included CCRT. Two other studies [15,16] included CCRT and SCRT but did not evaluate the HR.
4. In Harris’s study [15], the radiotherapy course length ranged from 3 to 9 weeks, which was too long a course and may have influenced the results for OS. The doses in other studies were generally between 50 and 70 Gy, with a mean of 60 to 65Gy, and had better concordance.

### Implications for clinicians and policymakers

Because of the limited number and design types of the original studies, we remain cautious about the superiority of IMRT to 3DCRT in OS and radiation pneumonitis the inferiority of IMRT to 3DCRT in radiation oesophagitis until further high-level Cochrane evidence becomes available, particularly randomized trials. However, our study makes unique contributes to the literature by documenting that IMRT is not inferior to 3DCRT in OS and has advantages in reducing radiation pneumonitis, particularly in patients with a larger extent of disease, such as lymph node metastasis. In addition, IMRT may increase the incidence of radiation oesophagitis.

With growing pressure on health insurance providers, the high cost of IMRT for lung cancer treatment could be a considerable financial burden [34–36]. The cost of IMRT is much higher than 3DCRT. In some areas, such as Korea, national health insurance systems do not reimburse patients for IMRT. In poverty-stricken zones, the use of IMRT will impose a significant burden and may influence follow-up treatments after radiation.

Therefore, careful and reasonable decision-making becomes a crucially important issue for the optimal utilization of current technologies and resources. In practice, we should make decisions based on the patient’s condition. For patients with a larger disease extent or more lymph node involvement, we might choose IMRT.

### Implications for future research

Future studies should, as much as possible, adopt cohort studies or randomized trials and control for confounding factors, particularly the radiotherapy dose, CCRT/SCRT, chemotherapy agents, and targeting therapies, and include simultaneous analyses of PFS and LRC and toxicity assessments. Toxicity assessments should include all grades of radiation pneumonitis or oesophagitis to calculate the HR of toxicity.

## Conclusions

Our meta-analysis determined that IMRT is not inferior to 3DCRT in the OS of NSCLC but, although it has advantages in reducing the incidence of radiation pneumonitis, increases the incidence of radiation oesophagitis, although the number of original studies was limited. The decision to use IMRT for the treatment of NSCLC should be based on the patient’s condition. Future studies, particularly randomized trials, are needed to demonstrate superior outcomes of IMRT compared to 3DCRT in the treatment of NSCLC.

## Supporting Information

S1 Table PRISMA 2009 Checklist for this article. (DOC)
